# The Dynamic Features of Lip Corners in Genuine and Posed Smiles

**DOI:** 10.3389/fpsyg.2018.00202

**Published:** 2018-02-21

**Authors:** Hui Guo, Xiao-Hui Zhang, Jun Liang, Wen-Jing Yan

**Affiliations:** ^1^Wenzhou 7th People's Hospital, Wenzhou, China; ^2^Institute of Psychology and Behavior Sciences, Wenzhou University, Wenzhou, China

**Keywords:** dynamic features, genuine smiles, posed smiles, lip corners, OpenFace

## Abstract

The smile is a frequently expressed facial expression that typically conveys a positive emotional state and friendly intent. However, human beings have also learned how to fake smiles, typically by controlling the mouth to provide a genuine-looking expression. This is often accompanied by inaccuracies that can allow others to determine that the smile is false. Mouth movement is one of the most striking features of the smile, yet our understanding of its dynamic elements is still limited. The present study analyzes the dynamic features of lip corners, and considers how they differ between genuine and posed smiles. Employing computer vision techniques, we investigated elements such as the duration, intensity, speed, symmetry of the lip corners, and certain irregularities in genuine and posed smiles obtained from the UvA-NEMO Smile Database. After utilizing the facial analysis tool OpenFace, we further propose a new approach to segmenting the onset, apex, and offset phases of smiles, as well as a means of measuring irregularities and symmetry in facial expressions. We extracted these features according to 2D and 3D coordinates, and conducted an analysis. The results reveal that genuine smiles have higher values for onset, offset, apex, and total durations, as well as offset displacement, and a variable we termed Irregularity-b (the *SD* of the apex phase) than do posed smiles. Conversely, values tended to be lower for onset and offset Speeds, and Irregularity-a (the rate of peaks), Symmetry-a (the correlation between left and right facial movements), and Symmetry-d (differences in onset frame numbers between the left and right faces). The findings from the present study have been compared to those of previous research, and certain speculations are made.

## Introduction

Among the various interpersonal social signals, facial expressions are one of the most frequently used to express social intentions. In human social interactions, the smile is the most common. A smile typically reflects a happy mood (i.e., a genuine smile), but people often disguise their smiles according to the situation. For example, in greetings and conversations, people may deliberately smile out of politeness (Ambadar et al., 2009; Hoque et al., 2011; Shore and Heerey, 2011). In situations where an individual intends to deceive another, the liar may deliberately display a pleasant expression to mask their ill intent and enhance their credibility, in order to appear amiable (Hoque et al., 2011). Smiles that are not elicited via the genuine subjective experience of happiness are often called deliberate, posed, false, social, polite, or masking smiles (Ekman and Friesen, 1982; Scherer and Ellgring, 2007; Krumhuber and Manstead, 2009; Mavadati et al., 2013; Gutiérrezgarcía and Calvo, 2015). For the remainder of this article, we will refer to masked or deliberate smiles as “posed” smiles, while spontaneous and authentic smiles will be referred to as “genuine.” This will assist us in better clarifying the numerous concepts we will address.

### Dynamic feature differences between genuine and posed smiles

Previous studies have investigated the differences between genuine and posed smiles, and reported several key indicators and features that may help to differentiate between the two, such as the Duchenne marker, duration, intensity, symmetry, and smoothness.

#### Duchenne markers

Duchenne markers are important features of genuine smiles. According to the Facial Action Coding System (Ekman et al., 2002), a Duchenne smile consists of Action Units (AU) AU6 and AU12. AU6 indicates a contraction of the orbicularis muscle, which represents the lifting of the cheek muscles and leads to the formation of crow's feet (Ekman et al., 1988). AU12 indicates a contraction of the zygomaticus muscle, which extends the corners of the mouth sideways and lifts the lip corners up to form a prominent U-shape or happy face. According to previous studies, a smile should only be considered genuine when both AU6 and AU12 occur simultaneously (Ekman, 2006). Frank and Ekman (1993) speculated that most people are able to voluntarily control AU12; in contrast, only a small percentage of people (20%) are able to voluntarily regulate AU6. Hence, it was concluded that AU6 would be a better indicator of a genuine smile, and this later became known as the “Duchenne marker.”

Researchers have often observed these Duchenne markers in individuals presented with pleasant stimuli (Ekman et al., 1990; Soussignan and Schaal, 1996) and/or in those providing self-reports that indicate a pleasant state of mind (Ekman et al., 1988; Frank et al., 1993). However, there is still significant debate regarding whether AU6 can be considered the gold standard, because other researchers have observed individuals behaviorally controlling their facial expressions and voluntarily expressing Duchenne smiles, even in the absence of pleasant or happy emotions. Krumhuber et al. (2009) found that in genuine smiles, the ratio of Duchenne smiles to non-Duchenne smiles is 70–30%, respectively, while for posed conditions it was 83–17%, respectively. Several other studies reported observing high proportions of posed smiles that fit the Duchenne smile criteria. For example, it was argued in one study that 56% of smiles fitting the posed smile condition met the criteria for Duchenne smiles (Schmidt et al., 2006). Other researchers have observed up to 60% (Gosselin et al., 2002), 67% (Schmidt and Cohn, 2001), and 71% (Gunnery et al., 2013) of posed smiles fitting the Duchenne smile criteria. These findings suggest that the standards for differentiating and recognize genuine smiles may indeed require more than Duchenne markers.

Other studies have also questioned whether Duchenne smiles actually indicate whether an individual is experiencing a pleasurable or happy emotion. It was reported that smiles fitting the Duchenne smile criteria were observed when participants watched videos designed to elicit negative emotions (Ekman et al., 1990), as well as when they failed in a game (Schneider and Josephs, 1991). These findings suggest that AU6 is more likely to reflect emotional intensity than pleasant or positive emotions, a speculation that is supported by studies that observed negative expressions such as sadness and pain also incorporating AU6 (Bolzani-Dinehart et al., 2005). Krumhuber and Manstead (2009) suggested that Duchenne markers might indicate intensity rather than pleasant or positive emotions. In their study, participants were asked to score the force of Duchenne and non-Duchenne smiles. The results revealed that the intensity score for a “Duchenne smile” (on a scale of 1 to 5, with 1 representing very weak and 5 indicating very strong) was significantly higher (*M* = 3.11) than a “non-Duchenne smile” (*M* = 0.97). Based on these and other findings, it would seem that Duchenne markers alone might not be a sufficient indicator of genuineness in a smile; hence, practitioners and layperson alike have begun to seek other indicators that could better aid in differentiating between genuine and posed smiles.

#### Duration

Genuine and posed smiles tend to differ in duration. According to the Investigator's Guide for FACS, onset time is defined as the length of time from the start of a facial expression to the moment the movement reaches a plateau where no further increase in muscular action can be observed. Apex time is the duration of that plateau, and offset time is the length of time from the end of the apex to the point where the muscle is no longer in action (Ekman et al., 2002). The total duration of a genuine smile can range from 500 to 4,000 ms, while posed smiles can be either longer or shorter (Ekman and Friesen, 1982). Previous studies have reported differences in duration for the various phases of genuine and deliberate expressions. Accordingly, an expression can typically be divided into the onset, apex, and offset phases, wherein the subjective experiences of emotions elicit and form the onset. If the emotional experience is sufficiently intense, it creates and possibly prolongs the apex phase. As the subjective experience of the emotion subsides, the activated facial muscles gradually return to a relaxed state or neutral expression, which marks the end of the offset phase and typically signals the end of the facial expression. Genuine smiles tend to have a slower onset speed and longer onset duration than posed smiles (Hess and Kleck, 1990; Schmidt et al., 2006, 2009). According to Ekman (2009), very brief (<0.5 s) or very long (>5 s) durations of expression occur more often in deliberate rather than spontaneous expressions. As for onset phase duration, genuine smiles can range between 0.5 and 0.75 s. Solitary spontaneous smiles have an average onset duration of 0.52 s, and spontaneous smiles produced in a social context average an onset duration of 0.50, 0.59, and 0.67 s (Schmidt et al., 2003, 2006, 2009; Tarantili et al., 2005).

Speed is yet another commonly investigated parameter. In previous research, the smile samples investigated were generally of high intensity or fully expressed. Schmidt et al. (2006) found that onset and offset speeds and offset duration were all greater in posed smiles. Likewise, Cohn and Schmidt (2004) reported that posed smiles had faster onsets. Finally, Schmidt et al. (2006) observed greater onset and offset speeds, amplitude (displacement) of movement, and offset duration in posed smiles.

#### Intensity

According to FACS and numerous previous studies, the intensity of a facial expression ranges from A (a trace) to E (a full-blown expression); an alternative scale ranges from 1 (of weak intensity) to 5 (of very strong intensity). Accordingly, Krumhuber et al. (2009) investigating the intensity of Duchenne markers in Duchenne and non-Duchenne smiles, reporting that the intensity (*M* = 3.07) of the Duchenne smiles was greater than that of the non-Duchenne smiles (*M* = 1.77). This finding was replicated in research conducted by Krumhuber and Manstead (2009), where participants either smiled spontaneously in response to an amusing stimulus (a spontaneous condition) or were instructed to pose a smile (a deliberate condition). Coders were then tasked to rate and record the highest intensity AU12 motions for each facial expression. The results revealed that Duchenne smiles were rated as more intense (*M* = 3.11) than non-Duchenne smiles (*M* = 0.97). Interestingly, this study also indicated that deliberate Duchenne smiles were rated as more intense (*M* = 3.37) than spontaneous Duchenne smiles (*M* = 2.85). These finding suggests that individuals who fake smiles are capable of behaviorally controlling their facial movements, and tend to express exaggerated smiles that are more intense than genuine smiles. In line with these findings, Schmidt et al. (2006) observed that the amplitude (displacement) of movement was greater in deliberate smiles.

#### Symmetry

Genuine and posed smiles may also have different features with regards to symmetry (Frank and Ekman, 1993; Frank et al., 1993). When asymmetries occurred in posed smiles, they were usually stronger on the left side of the face. Ekman et al. (1981) videotaped children spontaneously making happy faces that were elicited by jokes or encouragement; these were then compared to posed happy faces. Smiles formed in response to watching an amusing film were nearly always symmetrical (96%); expressions in response to negative emotions from watching unpleasant films were, for the most part, also symmetrical (75%). A meta-analysis revealed that this asymmetry was stronger for posed than spontaneous emotional expressions (Skinner and Mullen, 1991). A later review (1998) of 49 experiments shows that posed and spontaneous expressions did not differ in the direction of facial asymmetry, unlike clinical observations indicating that spontaneous expressions showed more bilaterality Borod et al. (1998). A more recent review (Powell and Schirillo, 2009) described non-clinical studies and suggested that there actually is facial asymmetry, with emotions being expressed more on the left side of the face than on the right in both spontaneous and posed expressions. However, several studies (Schmidt et al., 2006, 2009; Ambadar et al., 2009) utilized computer vision techniques to measure the displacement of action units associated with smiling, and observed no differences in the asymmetry of intensity (i.e., the amplitude) between genuine and posed expressions.

Regarding temporal asymmetries, Ross and Pulusu (2013) employed high-speed cameras to isolate time features and examine asymmetries in genuine and posed expressions. Posed expressions overwhelmingly originated on the right side of the face, whereas spontaneous expressions began most often on the left. In the upper half of the expressions, this pattern was particularly stable. In another study, however, Schmidt et al. (2006) found no differences between genuine and posed smiles in terms of asymmetries in onset or offset duration. A number of other researchers also found no difference.

#### Irregularity (smooth)

The degree of irregularity in genuine and posed smiles may also differ. Some facial expressions are very irregular; an apex may be steady or there may be noticeable changes in intensity before the offset phase begins (Ekman et al., 2002). The degree of irregularity refers to whether there are pauses or discontinuous changes in the phases of the expression (e.g., onset, apex, offset). Although this varies with the particular social circumstances, the onset of a deliberate expression will often be more abrupt than that of a spontaneous expression (Ekman, 2006). Hess and Kleck (1990) defined irregularity as the number of onsets and offsets throughout the entirety of the expression, and found that genuine expressions are more regular than those that are posed. Frank et al. (1993) used a different definition, smoothness, in their study. Smooth refers to the degree of positive correlations among the durations of the onset, apex, offset, and complete expression. This study found that genuine smiles (those with AU6) were smoother than posed expressions.

### Analyzing facial expressions using computer vision techniques

Because an historic lack of easy-to-use quantitative analysis tools (Frank et al., 2009), only a handful of studies on the dynamic characteristics of expression (such as duration, velocity, smoothness, motion symmetry, synchronization of different parts, etc.) have been conducted. With the development of computer vision and pattern recognition techniques, researchers have begun to employ new analysis tools to further study facial expressions. For example, by tracking the various parts of the face over time, they have been able to witness gradual changes in intensity for each phase, the symmetry of synchronization of left and right movements, and so on.

Considering the difficulties in manual coding using FACS, computer researchers have been working on developing new and better face analysis tools. The analysis of facial expressions generally involves three steps: detecting the face in a picture or video, extracting the facial features, and recognizing and classifying those features. The field of computer vision focuses on how to accurately classify different expressions (Pantic and Patras, 2006; Sebe et al., 2007) and AU (Cohn and Sayette, 2010; Littlewort et al., 2011; Wu et al., 2012; Mavadati et al., 2013). From a psychological perspective, researchers are more interested in how particular dynamic features distinguish different smiles. Therefore, the focus is on the feature extraction method and quantitative analysis of the facial movement. Here, feature extraction refers to the use of computers to extract image information, in order to determine whether the points in each image belong to a particular image feature. In other words, this process looks for image information (such as edges, corners, textures, etc.) that is specific to the original feature (a number of pixels). Feature extraction methods are mainly divided into two categories: geometric feature-based approaches and appearance-based methods (Tian et al., 2011). A system based on geometric features extracts the shape and position of the facial composition (such as the mouth, eyes, eyebrows, nose, etc.); an appearance-based methodology uses visual features to represent the object. These two types of processes have different levels of performance in extracting different features, but the merits of the performances are uncertain.

Schmidt et al. (2006) used the CMU / Pitt Automatic Facial Analysis System (AFIA) to measure the movement characteristics of the large zygomatic muscle during genuine and posed smiles. This system automatically fits the landmarks on the first frame of the video clip, and then uses the Lucas-Kanade optical flow (OF) algorithm to track the feature points, after correcting for head motion. The algorithm tracks a pixel on the first frame of the image and determines the position of that pixel on subsequent images, in order to determine the pixel's coordinate changes. The intensity is defined by the moving distance of the feature points, divided by the width of the mouth. With further calculations, the duration, displacement, and velocity of the mouth movement can all be quantified. The results of Schmidt et al. (2006) indicate that compared to posed expressions, mouth movement during onset and offset were shorter and faster in genuine smiles, but there was no difference in symmetry between the two.

Dibeklioǧlu et al. (2012) extracted 25 descriptors (features) to train a classifier, as is common practice with computer vision researchers, in order to distinguish genuine from posed smiles; these descriptors included duration, duration ratio, maximum and mean displacements, the SD of the amplitude, total and net amplitudes, amplitude ratio, maximum and mean speeds, maximum and mean accelerations, net amplitude, duration ratio, and left/right amplitude difference for three different face regions (eyes, cheeks, and mouth). After the feature extractions, the researchers trained a classifier that attempted to recognize whether a given video was genuine or posed.

Yan and Chen (in press) tried to quantify micro-expressions using the Constraint Local Model (CLM) and Local Binary Pattern (LBP) methods. The CLM process detects 66 feature points for each face image and tracks the movement and distance for each of these landmarks. These feature points are distributed on the contours of the head, eyes, nose, and mouth. The dynamic features of these landmarks are then described by calculating their position changes over time. Based on the feature points, Yan and Chen (in press) divided the facial area into 16 areas of interest (such as the insides of the eyebrows, which is Interest Area 1), and extracted their texture features using LBP. By comparing the correlations among the textures of the first and subsequent frames, the motion features could be described. The researchers then tested the effects of these two feature extraction methods on 50 micro-expressions, finding that they were similar to manual coding when determining the peak frame.

### The aim of this work

Previous research has considered the Duchenne mark (AU6), duration, symmetry, irregularity, and other clues in order to investigate the differences between genuine and posed smiles. However, while some indicators have inspired a fairly stable consensus, others have continued to be controversial. We employed a newly-developed analytical tool to investigate specific movements of the mouth and lip corners, which are the most prominent and easily posed in a smile. In Dibeklioǧlu et al. (2012), 25 features were considered. Many of these features were difficult to explain from a psychological perspective. Based on this previous research, we extracted duration, speed, intensity, symmetry, and irregularity.

We conducted feature extractions to produce 2D and 3D coordinates with OpenFace, in order to investigate how certain dynamic features of the lip corners differed between genuine and posed smiles. Overall, we hypothesized that genuine smiles would be of longer duration, slower speed, and lower intensity; we also explored the differences in irregularity and symmetry between the two types of smiles.

## Methods

### Materials

We used the UvA-NEMO Smile Database (Dibeklioǧlu et al., 2012) to analyze the dynamics of genuine and posed smiles of enjoyment. The database consists of 1,240 smile videos (597 spontaneous and 643 posed) obtained from 400 subjects (185 female and 215 male), making it the largest smile database in the literature, to date. The ages of the subjects varied from 8 to 76 years, with 149 subjects being younger than 18 years (offering 235 spontaneous and 240 posed smiles). Of the total, 43 subjects did not have spontaneous smile samples and 32 had no posed smiles. The videos are in RGB color and were recorded at a resolution of 1,920 × 1,080 pixels, at a rate of 50 frames per second, and under controlled illumination conditions.

For the posed smiles, each subject was asked to posture an enjoyment smile as realistically as possible, after being shown a sample video of a prototypical smile. This differed from the samples in Schmidt et al. (2006), where the spontaneous smiles were not the result of any specific elicitation procedure. These genuine smiles of enjoyment were elicited by a set of short, funny video segments shown to each subject for approximately 5 min. The mean duration of the spontaneous and posed smile segments was 3.9s (σ = 1.8), and the average interocular distance from the database was approximately 200 pixels. The segments all began and ended with neutral or near-neutral expressions. This is considered a well-established database that not only contains a large sample size, but also offers a well-designed lab situation and well-set elicitation approach.

### Analysis tool: openface

OpenFace (Baltrusaitis et al., 2016) is not only the first open source tool for facial behavior analysis, it demonstrates state-of-the art performance in facial landmark detection (Baltrusaitis et al., 2013), head pose tracking, AU recognition (Wood et al., 2015) and eye gaze estimation (Wood et al., 2015). The source code can be downloaded here^1^. OpenFace 0.3.0 provides 2D and 3D spatial landmarks for analyzing faces. In this study, the results from a variety of different landmark systems are examined and discussed.

### Design

The independent variable for this research was authenticity: genuine / posed. The dependent variables included: duration, speed, intensity, symmetry, and irregularity (see Table 1 for details). In the database, each participant provided at least one trial for a genuine or posed condition, so this was considered a within-subject design.

**Table 1:** Definitions of the features extracted for a single facial expression.

**Feature**	**Definition**
Duration	$\frac{F(S^{+})}{F(S)},\frac{F(S^{=})}{F(S)},\frac{F(S^{-})}{F(S)}$
Duration Ratio	$\frac{F\left(S^{+}\right)}{F\left(S\right)}\;$, $\frac{F\left(S^{=}\right)}{F\left(S\right)}$, $\frac{F\left(S^{-}\right)}{F\left(S\right)}$
Displacement	$max(D),D_{offset}$
Speed	$\frac{\sum D^{+}}{F(D^{+})},\frac{\sum D^{-}}{F(D^{+})}$
Irregularity	$\frac{P}{F(S)/r},SD(D^{=})$
Symmetry	$\begin{array}{l} Cor(D_{L},D_{R}),M(D_{R}^{=})-M(D_{L}^{=}),F_{R-onset}-F_{L-onset},|F_{R-onset}\\ -F_{L-onset}| \end{array}$

In Table 1, *S* indicates a complete smile. The signals are symbolized with a super-index, and (+), (=), and (–) denote the segments of onset, apex, and offset, respectively. For example, *S*^+^ pools the onset segments for one smile, *N* defines the number of frames in a given signal, and *r* is the frame rate of the video. *D* defines the displacement (the difference in amplitudes between the fiducial and selected frames) of a given signal. *D*_*L*_ and *D*_*R*_ are the displacements of the left and right lip corners, respectively. *P* defines the number of peaks (an onset and offset form one peak, but only displacement differences larger than 10% *D*_*max*_ between adjacent peaks and valleys were filtered out). We measured the offset displacement (*D*_*offset*_) because from our observations it seemed that spontaneous smiles usually ended with the trace of a smile. Therefore, we hypothesized that the displacement in offset frames would be larger in spontaneous smiles than in those that were posed.

In addition to conventional features such as duration, displacement, and speed, we also examined other dynamic elements such as irregularity and symmetry. For irregularity, we used two indicators: Irregularity-a and Irregularity-b. Irregularity-a defined the number of peaks per second. This was similar to Hess and Kleck's (1990) method, where the onsets and offsets for each facial expression were counted as irregularities. Irregularity-b defined the standard deviation (SD) values for the apex displacements. SD was used to quantify the amount of variation or dispersion of a set of data values. We also used SD to measure changes in the apex phase. If the apex phase was just a plateau, the SD was close to zero; when there was substantial fluctuation in the apex phase, the SD was large. However, it would not have been appropriate to use SD to measure the onset and offset phases, because it would have made it difficult to find any psychological meaning.

For symmetry, we explored four different methods. For simplicity, we labeled them a, b, c, and d. In terms of the lip corners, Symmetry-a, *Cor* (*D*_*L*_, *D*_*R*_), defined the Pearson correlation coefficient, Symmetry-b described the mean displacement differences for the apex phase, and Symmetry-c reflected the onset frame differences from a temporal perspective. Symmetry-d denoted the absolute value of Symmetry-c. Here Symmetry-c and Symmetry-d are the “reversed scoring” index, the larger, the more asymmetric.

### Procedure for using 3D landmarks

#### 3D pose correction

OpenFace uses the recently proposed Conditional Local Neural Fields (CLNF) (Baltrusaitis et al., 2013) for facial landmark detection and tracking. Sixty-eight 3D landmarks are detected in each frame, and the 3D coordinates for each landmark are generated. In addition, OpenFace provides 3D head pose estimations, as well as roll (θ*z*), yaw (θ*y*), and pitch (θ*x*) rotations.

Since head movements may occur along with facial movements, it was essential remove (or control for) the influence of the head pose. In this research, head pose estimations for three directions (or rotations) were transformed into a rotation matrix, and each landmark in the 3D space was corrected by post-multiplying the corresponding rotation matrix.

(1) $$\begin{array}{r} {l_{i}^{t}}^{\prime }=l_{i}^{t}R_{x}\left(-\theta_{x}\right)\;R_{y}\left(-\theta_{y}\right)R_{z}\left(-\theta_{z}\right) \end{array}$$

where *l*_*i*_ is the aligned landmark and *R*_*x*_, *R*_*y*_, and *R*_*z*_ denote the 3D rotation matrices for the given angles. Moreover, to control for the influence of head translation (left–right or up–down movement), we selected one stable point, landmark 34 (indicating the inner nostril), and subtracted the other landmark coordinates from it. In previous studies, inner eye corners were often considered stable and used as the reference. However, in most face alignment tools, inner eye corner landmarks change sharply when eyes blink. Therefore, such a reference is actually unsuitable when there are eye blinks in the facial expression.

(2) $$\begin{array}{r} {l_{i}^{t}}^{\prime \prime }={l_{i}^{t}}^{\prime }-{l_{34}^{t}}^{\prime } \end{array}$$

#### Displacement measurement

After correcting the head pose rotation and translation, we proceeded to divide the smiles into the onset, apex, and offset phases. This phase segmentation relied on the pattern of movement in the lip corners, which is quite conspicuous in a smile (the lip corners pull backward and upward). By tracking the lip corners over time, we were able to gain the lip corner coordinates in the world coordinates for each frame. We could then calculate the displacement of the lip corners.

There were several possible ways to describe the displacement of lip corner movements: (1) the initial center point could be calculated as the midway position between the lip corners in the initial frame. This initial center point is then recalculated automatically in each frame, relative to the stable inner eye corner feature points, allowing for accurate measurement in cases of small head movements. The pixel coordinates of the right lip corner in subsequent frames, relative to the initial center point of the lip corners in the initial frame, are then automatically obtained using the Lucas–Kanade algorithm for feature tracking (Lien et al., 2000). The displacement of the right lip corner is considered the indicator of the lip movement. The displacement is standardized for the initial width of each participant's mouth in the initial image.

(3) $$\begin{array}{r} D_{lip}\left(t\right)\;=\rho \left(\frac{l_{r}^{t}+l_{l}^{t}}{2},l_{r}^{t}\right) \end{array}$$

where $l_{r}^{t}$ and $l_{l}^{t}\;$denote the coordinates of the right and left lip corners in frame *t*, respectively, and ρ is the Euclidean distance between the given points.

(2) Dibeklioǧlu et al. (2012) estimated the smile amplitude as the mean amplitude of the right and left lip corners, normalized by the length of the lip. Let *D*_*lip*_*(t)* be the value of the mean displacement signal of the lip corners in frame *t*. This can be estimated as:

(4) $$\begin{array}{r} D_{lip}\left(t\right)=\frac{\rho \left(\frac{l_{49}^{1}+l_{55}^{1}}{2},l_{49}^{t}\right)+\rho \left(\frac{l_{49}^{1}+l_{55}^{1}}{2},l_{55}^{t}\right)}{2\rho \left(l_{49}^{1}\;,l_{55}^{1}\right)} \end{array}$$

where $l_{i}^{t}\;$denotes the 3D location of the *i-th* point (in this research, points 49 and 55 indicated the right and left lip corners, respectively) in frame *t*, and ρ is the Euclidean distance between the given points.

(3) Lip corners movements can be calculated according to changes in each landmark location, across time.

(5) $$\begin{array}{r} D_{lip}\left(t\right)=\frac{\rho \left(l_{49}^{1},l_{49}^{t}\right)+\rho \left(l_{55}^{1},l_{55}^{t}\right)}{2} \end{array}$$

We used this simple calculation in this research because: (1) the initial middle point was unsteady, due to the movement of the lip corners (e.g., when the two lip corners experienced unbalanced changes, the middle point of the lips deviated); (2) we compared genuine with posed smiles using a within-subject design, which meant that the length of the mouth for the given participant was the same, and thus there was no need for normalization; (3) with head pose (rotation and translation) corrections, the faces from different frames were aligned, and thus the displacement of the lip corners could be calculated according to the coordinates of the lip corners.

#### Phase segmentation

**Figure 1:**
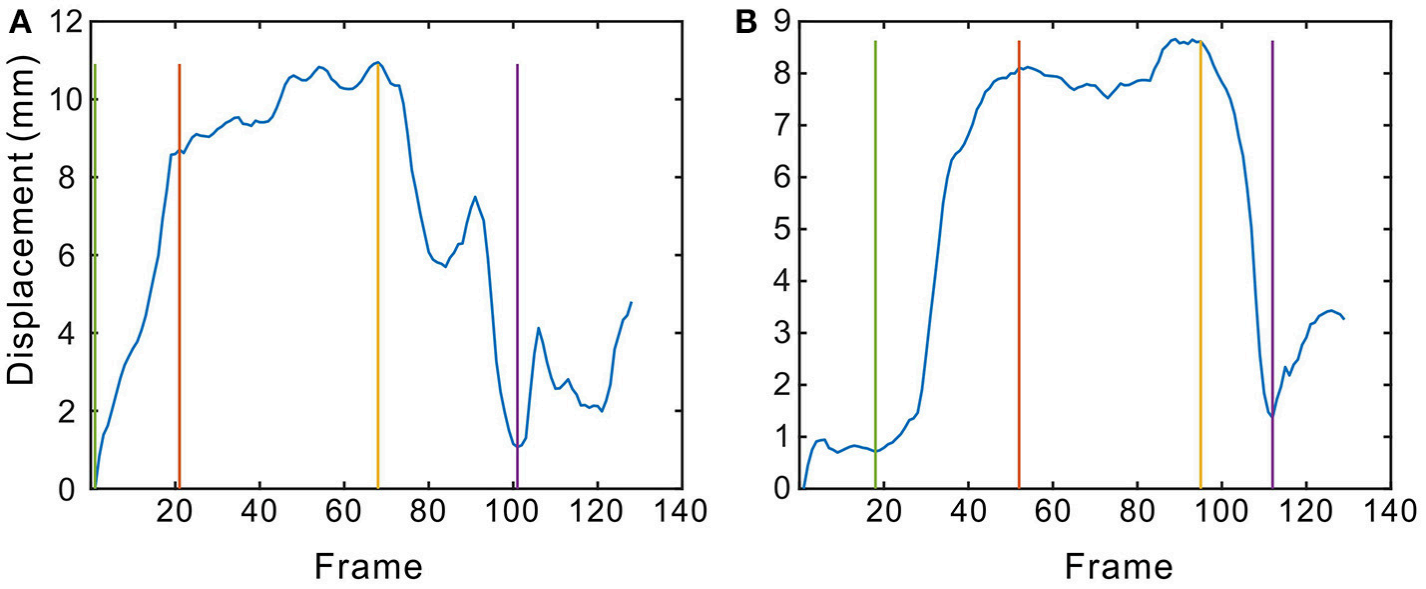
The displacement of the lip corner movements across time. **(A,B)** are two examples. The blue curve indicates that the lip corner displacement changes across time, while the green line denotes onset, the red and yellow lines reference the boundary of the apex phase, and the purple line highlights the offset. The steps taken to complete this segmentation are described in section Phase Segmentation.

Based on the lip corner displacement, we attempted to segment the smiles into three phases: onset, apex, and offset. As Dibeklioǧlu et al. (2012) mentioned, the longest continuous increase in *D*_*lip*_ is defined as the onset phase. Similarly, the offset phase is the longest continuous decrease in *D*_*lip*_. The phase between the last frame of the onset and the first frame of the offset is the apex. This is a very easy and effective way of segmenting the different phases. However, when the smile movement is not very regular (usually displayed as small peaks in the curves, as seen in Figure 1), the segmentation method (the longest continuous increase / decrease) may not be sufficiently accurate. According to FACS, an apex may be steady or there may be noticeable fluctuations in intensity before offset begins. Hess and Kleck (1990) calculated all onset, apex, and offset durations for a single facial expression by the naked eye, with subjective definitions of all three phases. If the facial expression was not smooth (i.e., regular), there were several possible onset durations. In this research, we used the UvA-NEMO Smile Database, where only a single smile was contained in each video episode. We segmented only single smiles with one set of onset, apex, and offset phases.

This study attempted to simplify phase segmentation while considering the irregularity of facial movements. To do so, we proposed a new method for segmenting the phases, as follows:

1. Smooth the displacement of the lip corners across time using a moving average filter, at a window length of 3.
2. Find all peaks (there are still peaks or bulges, even after smoothing), including the highest (maximum displacement of lip corners in a given smile, *D*_*max*_).
3. Define the apex phase as the regions between peaks that are higher than 70% of the *D*_*max*_. Sometimes there is only one peak that is higher than 70% of the *D*_*max*_ (this is actually the highest peak); in such cases, the apex phase consists of only one frame.
4. Define the onset phase as the region between the onset frame and onset-apex boundary. For all of the valleys before the apex phase, the valley that is nearest the apex phase where the displacement is less than 20% of the *D*_*max*_ is the onset frame. If there is no such valley, the lowest displacement frame before the apex phase is the onset frame.
5. Define the offset phase as the region between the offset frame and apex-offset boundary. For all of the valleys after the apex phase, the valley nearest the apex phase where the displacement is less than 20% of the *D*_*max*_ is the offset frame. If there is no such valley, the lowest-displacement frame after the apex phase is the offset frame.

Peaks: *D*_(*i*−1)_–*D*_*i*_ < 0 and *D*_(*i*+1)_–*D*_*i*_ ≥ 0

Valleys: *D*_(*i*−1)_–*D*_*i*_ > 0 and *D*_(*i*+1)_–*D*_*i*_ ≤ 0

where D_*i*_ indicates the displacement of the *i-th* frame.

### Procedure for using 2D landmarks

The procedure for the 2D landmark system is quite similar to that of the 3D landmark system. Sixty-eight 2D landmarks were detected in each frame. We selected one stable landmark, landmark 34 (indicating the inner nostril) and subtracted the other landmark coordinates from it. Lip corner movements were calculated according to Equation (3). Based on the level of lip corner displacement, we attempted to segment the smiles into three phases: onset, apex, and offset. Then, the dynamic features of the lip corners during the smiles were extracted.

## Data analysis and results

### Using the 3d landmark system

After analyzing 1,240 video episodes of smiles from 400 subjects, we extracted 22 features for each smile. Certain smile video episodes were removed based on the following two conditions: (1) the offset phase displacement was larger than that of the apex phase; and (2) the offset phase was less than 0.2 s. These smile video episodes were removed because they tended to exhibit complex facial expressions or be prone to incorrect manual segmentation. As a result, 124 samples were excluded. We aggregated genuine and posed conditions for each subject. Those with only spontaneous or deliberate smiles were also removed. In the end, 297 subjects exhibiting both smile conditions were included for further analysis.

The *p*-value is highly affected by the sample size. In particular, when the sample size approaches 250, the difference / effect is statistically significant regardless of the alpha level (Figueiredo Filho et al., 2013). Also, as Hair et al. (1998) said, “by increasing [the] sample size, smaller and smaller effects will be found to be statistically significant, until at very large sample sizes almost any effect is significant.” Due to the large sample size in this study, the F-value could have been inflated, and thus the *p*-values easily influenced. Therefore, we set a strict cut-off point at *p* ≤ 0.01, and placed more emphasis on the effect size. Richardson (2011) argued that *partial η*^2^ values of 0.0099, 0.0588, and 0.1379 could serve as benchmarks for small, medium, and large effect sizes, respectively.

A repeated measures multivariate analysis of variance (MANOVA) was conducted to examine the effect of the independent variable (authenticity: genuine / posed) on the combined dependent variables (facial features).

A one-way (authenticity: genuine / posed) repeated measures MANOVA was conducted for each participant's facial features. These analyses confirmed that there was a significant multivariate effect for authenticity [*F*_(15, 282)_ = 37.126, *p* < 0.001, partial η^2^ = 0.664]. Within-group univariate analyses indicated no differences between the genuine and posed conditions for the following five dependent variables: onset, offset, and apex ratios, Symmetry-b, and Symmetry-c. Significant differences between genuine and posed conditions were observed for the remaining 12 dependent variables [*F*_(1, 296)_ ≥ 8.343, *p* ≤ 0.004, partial η^2^ ≥ 0.027]. The onset, offset, apex, and total durations, as well as the offset and standard apex displacements were observed to be significantly higher in the genuine condition. Onset and offset speeds, irregularity-a (rate of peaks), Symmetry-a, and Symmetry-d (smaller values means less asymmetric) were all observed to be significantly lower in the genuine condition. The means, *SD, F, p*, and partial η^2^ values are all shown in Table 2.

**Table 2:** Descriptive and inferential statistics for genuine and posed smiles from the 3D approach.

	**Genuine**		**Posed**		***F***	***P***	**Partialη^2^**
	***M***	***SD***	***M***	***SD***			
Onset Duration	0.93	0.55	0.57	0.24	130.375	<0.001	0.306
Offset Duration	1.10	0.89	0.68	0.34	60.481	<0.001	0.170
Apex Duration	2.97	1.55	1.84	0.74	138.446	<0.001	0.319
Total Duration	5.00	1.98	3.09	0.82	271.829	<0.001	0.479
Onset Ratio	0.20	0.09	0.19	0.08	2.889	0.090	0.010
Offset Ratio	0.23	0.12	0.22	0.10	0.259	0.611	0.001
Apex Ratio	0.57	0.15	0.59	0.12	1.966	0.162	0.007
Offset Displacement	3.04	1.58	2.62	1.22	13.752	<0.001	0.044
Max Displacement	12.19	3.43	12.97	3.05	10.339	0.001	0.034
Onset Speed	13.63	7.43	23.73	10.34	244.854	<0.001	0.453
Offset Speed	9.94	6.35	16.83	9.94	116.720	<0.001	0.283
Irregularity-a	0.78	0.43	0.86	0.36	8.343	0.004	0.027
Irregularity-b	0.89	0.48	0.66	0.38	46.802	<0.001	0.137
Symmetry-a	0.92	0.11	0.96	0.06	32.834	<0.001	0.100
Symmetry-b	2.80	1.98	2.74	2.00	0.209	0.648	0.001
Symmetry-c	−1.28	5.93	−0.49	3.85	3.997	0.046	0.013
Symmetry-d	3.78	5.04	2.23	3.35	22.154	<0.001	0.070

*The order of the variables corresponds to the variable definitions in Table 1*.

### Using the 2d landmark system

The data removal criteria were discussed in section Using the 3D landmark system. As a result of this procedure, 96 samples were excluded. All subjects who only had either genuine or posed smiles in the database were removed. The result was that 302 subjects with both conditions were included for further analysis. A repeated measures multivariate analysis of variance (MANOVA) was conducted to examine the effect of the independent variable (authenticity: genuine / posed) on the combined dependent variables (the facial features).

One-way (authenticity: genuine / posed) repeated measures MANOVA analysis was conducted for the participants' facial features. These analyses confirmed that there was a significant multivariate effect for authenticity [*F*_(15, 287)_ = 43.636, *p* < 0.001, partial η^2^ = 0.695]. Within-group univariate analyses indicated no differences between the genuine and posed conditions for the following six dependent variables: onset, offset, and apex ratios, max displacement, Symmetry-b, and Symmetry-c. Significant differences between genuine and posed conditions were observed for the remaining 11 dependent variables [*F*_(1, 296)_ > 11.418, *p* ≤ 0.001, partial η^2^ ≥ 0.037]. Onset, offset, apex, and total durations, offset displacement, and Irregularity-b were observed to be significantly higher in genuine smiles. However, onset and offset speeds, Irregularity-a, Symmetry-a, and Symmetry-d were observed to be significantly lower for genuine smiles. The means, *SD*, F, *p*, and partial η^2^ values are all shown in Table 3.

**Table 3:** Descriptive and inferential statistics for genuine and posed smiles from the 2D approach.

	**Genuine**		**Posed**		***F***	***P***	**Partialη^2^**
	***M***	***SD***	***M***	***SD***			
Onset Duration	1.16	0.74	0.63	0.26	152.126	<0.001	0.336
Offset Duration	1.23	0.91	0.79	0.42	61.767	<0.001	0.170
Apex Duration	2.60	1.44	1.66	0.76	110.330	<0.001	0.268
Total Duration	5.00	2.00	3.08	0.82	259.848	<0.001	0.463
Onset Ratio	0.25	0.11	0.21	0.08	22.448	<0.001	0.069
Offset Ratio	0.26	0.13	0.26	0.12	0.439	0.508	0.001
Apex Ratio	0.50	0.16	0.52	0.15	5.842	0.016	0.019
Offset Displacement	6.97	4.74	5.36	3.85	24.125	<0.001	0.074
Max Displacement	31.46	9.61	31.87	9.32	0.376	0.540	0.001
Onset Speed	28.93	13.73	53.90	23.53	322.262	<0.001	0.517
Offset Speed	24.00	17.82	38.12	22.54	82.401	<0.001	0.215
Irregularity-a	0.59	0.30	0.74	0.31	43.528	<0.001	0.126
Irregularity-b	2.09	1.31	1.48	0.91	51.397	<0.001	0.146
Symmetry-a	0.81	0.24	0.87	0.22	11.418	0.001	0.037
Symmetry-b	0.49	8.12	−0.46	9.5	1.951	0.164	0.006
Symmetry-c	0.07	5.28	0.40	3.20	0.956	0.329	0.003
Symmetry-d	3.21	4.42	1.71	2.81	23.419	<0.001	0.072

*The order of the variables corresponds to the variable definitions listed in Table 1*.

## Discussion

Lip corner movement (AU12) is the core action unit of a smile; it is easily controlled and posed. This study considered the dynamic features of this action unit in both genuine and posed smiles. We extracted features using 2D and 3D coordinates, and found that the results were quite similar between the two approaches. Only the maximum amplitude was determined to be significant in the 2D method; this value was insignificant in the 3D approach. This research revealed that genuine smiles' onset, offset, apex, and total duration times were significantly longer than those of posed smiles. Genuine smiles also had higher offset displacement and Irregularity-b values (the *SD* of the apex phase) than did posed smiles. In contrast, posed smiles had faster onset and offset speeds. Furthermore, dynamic feature analyses of the left and right lip corners revealed that posed smiles were more asymmetrical than genuine smiles. These findings are discussed below, from a psychological perspective.

### Duration

Previous studies have reported that the duration of a facial expression can range from 0.5 to 4 s, and researchers have speculated that the duration of a posed smile is either longer or shorter (Ekman and Friesen, 1982) than one that is genuine. The facial expressions analyzed here lasted about 5 s for genuine smiles inspired by enjoyment, and approximately 3 s for smiles that were posed. Overall, the durations of spontaneous smiles were much longer during the onset, apex, and offset phases. These results are in line with previous findings (Hess and Kleck, 1990; Schmidt et al., 2006, 2009) that reported genuine expressions having slower onset speeds and, in general, longer total durations.

People seemed to be unaware of the longer durations of their spontaneous smiles, because even when asked to pose a smile as naturally as possible, the duration was nearly always much shorter. Thus, we inferred that in their minds, the prototypical pattern of a genuine smile was also much shorter. However, as perceivers, people are able to judge the authenticity of a smile by its duration. Duchenne smiles with longer onset and offset durations were judged to be more authentic than their shorter counterparts (Krumhuber and Kappas, 2005). Yet these researchers determined that the genuineness rating tended to decrease as a function of how long the smile was held at its apex. This conclusion contradicts our findings. One possible reason for this conflict may be that the stimuli in their study were synthesized faces, which could make the lasting-static apex phase wired.

Another explanation for people's inability to accurately simulate genuine smiles may be that they have a static pattern for smiling and ignore the more dynamic features. It's possible that they then pay more attention to morphological features such as the Duchenne marker, a notion that has repeatedly surfaced in previous research and been popularized by mass media (such as the BBC online test^2^). There may also be yet another explanation: the subjects can't hold their muscles in position for the proper length of time without the fuel provided by emotions.

To appear more genuine in times of enjoyment, people should begin at a slower pace, hold the smile longer, and fade at a reduced speed in terms of the Duchenne marker.

### Intensity

On the maximum displacement of smiles, the 3D approach showed that the intensity was higher in posed smiles, but the difference was insignificant with the 2D approach. It is important to note that this intensity was for lip corner movement and not for the smile itself. It seems counterintuitive that facial smiles elicited from strong emotions would be no more intense than those that are posed. In a posed condition, subjects must expend effort to pull their lips, perhaps even more than is actually needed and especially when displaying large smiles. This may be because they believe that large smiles feature wide lips and humans excel at lip control. Therefore, in a posed condition the intensity of the lip corners appears even larger than in a genuine smile. In genuine smiles and laughter, humans don't appear to pull their lip corners to their fullest possible extent. This conclusion echoes the common experiencing of cheek pain when laughing for an extended period of time, instead of pain emanating from the lip corners.

In this study, we also measured offset displacement, finding higher levels in genuine smiles. This verified our observation that spontaneous smiles usually ended with the trace of a smile. The expression of emotion involves a short and intense process that seems to have an additional, later influence on the expresser's mood. Previous researchers found that presenting emotional stimuli had an effect on subsequent behavior or processing (Barrett et al., 2016). Though strong emotions fade, relevant feelings or moods may linger. For example, after exultation, one does not instantly return to emotional neutrality; instead, a sense of happiness may remain. This type of trace or residual facial expression has not been properly studied. We hypothesize that emotionally elicited facial expressions generally leave a slight trace or a “long tail” after ending. This hint of genuine emotion may appear on the face for some time, even though it is too weak to discern. However, when compared with a neutral face, the subtle differences become obvious. This finding provides a new perspective from which to observe subtle emotional facial expressions.

### Symmetry

In this study, we attempted to measure symmetry from four indicators; two were based on intensity, and two on time features. We used a Pearson correlation coefficient to measure the intensity differences between the left and right lip corners (i.e., Symmetry-a). This was a pilot attempt at using a correlation coefficient as an indicator. The advantage was that this indicator considered all of the phases at once. Higher values reflected that the left and right corners moved more synchronically; however, such values didn't reveal intensity differences between the two sides. The asymmetries between genuine and posed smiles were found to be different. Though these results echoed much previous research (e.g., Ekman et al., 1981; Borod et al., 1998; Powell and Schirillo, 2009), Symmetry-a employed a different approach (i.e., the correlation coefficient of the intensity of the lip corners), suggesting that they actually cannot be compared directly with one another.

We also used Symmetry-b, similar to what was employed in previous studies, to calculate the mean displacement of the apex phase of the left and right lip corners. We employed intervals instead of a single point in order to keep this indicator reliable. However, the results showed no differences in this respect. For Symmetry-b, our results closely resembled those of a previous study (Schmidt et al., 2006), wherein no differences were observed between genuine and posed smiles with regards to the asymmetry of intensity (amplitude). It should be noted that different approaches were employed in measuring intensity; the other study used the maximum value of the amplitude (a single point) to indicate the intensity of a smile, while we took the mean value of the apex phase as the indicator. Considering that output values from computer vision algorithms are usually unsteady (i.e., they may feature a certain amount of noise), we used the mean instead of a single value.

Symmetry-c and Symmetry-d measured the onset frame differences from a temporal perspective. Symmetry-c reflected the onset frame gap between different smile types. Positive values indicated that the right side was stronger than the left, and negative values designated the reverse. In this study, no difference was found between genuine and posed smiles; this was unlike the results reported by Ross and Pulusu (2013), where posed expressions overwhelmingly originated on the right side of the face and spontaneous expressions on the left. If the positive–negative (left–right) direction was not considered (i.e., if the absolute value of the difference, Symmetry-d, was used) the results revealed that the genuine and posed smiles did differ, with the posed smiles being more asymmetrical (larger time gaps were observed between the left and right lip corners). Posed smiles were more asymmetrical; this echoed the results of many studies, though most observations were of intensity rather than onset time. Different from Ross and Pulusu's (2013) work that considered different types of asymmetry for different types of smiles and Schmidt et al.'s (2006) study that analyzed assymetrical differences between genuine and posed smiles, we observed another type of assymetry (the significance of Symmetry-d). This considerably complicated the temporal asymmetry.

### Irregularity

Only a very few studies have addressed irregularities in facial expressions. In our research, we used two indicators to describe irregularity. However, they did not support one another. Genuine smiles had greater values for Irregularity-a (the rate of peaks) and lesser values for Irregularity-b (the *SD* value for the apex phase). Irregularity-a was similar to the indicator used in Hess and Kleck (1990), and the results were consistent with their findings; posed smiles were more irregular.

Irregularity-b was a pilot indicator for this study. The values for Irregularity-b were larger for genuine smiles, indicating that during the apex phase, more changes were seen in genuine than in posed smiles. Because genuine emotions are not the same every time, genuine smiles tend to vary in strength, duration, and type. Even for smiles attributable to enjoyment, expressions may often differ in various ways. Conversely, in posed smiles, individuals may follow a prototypical pattern that results in the expressions being similar. Thus, the notion of irregularity requires further research.

### Some considerations regarding facial dynamics analysis with computer vision techniques

Over the past few years, there has been an increased interest in automatic facial behavior analysis. There are many algorithms currently available for analyzing facial movements, and specifically, lip corners. The current study is one of many that applies computer vision techniques to the analysis of nonverbal behavior.

OpenFace provided the 2D and 3D approaches we employed here. Traditionally, facial tracking has primarily been based on 2D methods, and these algorithms have matured. The 3D model in OpenFace is actually based on 2D images and does not actually include depth information from the camera. Instead, OpenFace uses a 3D representation of facial landmarks and projects them onto the image using orthographic camera projection. Therefore, the reliability and validity of the model used here needed to be further verified. The 3D model was able to extract head pose information (translation and orientation), in addition to detecting facial landmarks. This allowed us to accurately estimate the head pose once the landmarks were detected. With these considerations, we were able to employ two approaches that could be compared with one another.

The techniques are improving and as a result, accuracy will be enhanced. However, there are a variety of conflicting ideas regarding how to define features, not only in terms of the boundaries of phases, but also dynamic elements such as duration, speed, symmetry, and irregularity. Different groups have different definitions, which makes for inconsistencies in the literature. Therefore, we should be cautious when comparing results produced by different research groups.

## Author contributions

HG proposed the idea and gave suggestions in writing the paper; X-HZ designed the experiment and analyzed the data; JL analyzed the data; W-JY proposed the idea, designed the experiment, analyzed the data and wrote the paper.

### Conflict of interest statement

The authors declare that the research was conducted in the absence of any commercial or financial relationships that could be construed as a potential conflict of interest.

## Footnotes

^1^https://github.com/TadasBaltrusaitis/OpenFace.

^2^http://www.bbc.co.uk/science/humanbody/mind/surveys/smiles/index.shtml.
